# No increase in readmissions or adverse events after implementation of fast-track program in total hip and knee replacement at 8 Swedish hospitals: An observational before-and-after study of 14,148 total joint replacements 2011–2015

**DOI:** 10.1080/17453674.2018.1492507

**Published:** 2018-07-09

**Authors:** Urban Berg, Erik BüLow, Martin Sundberg, Ola Rolfson

**Affiliations:** 1 Department of Orthopaedics, Institute of Clinical Sciences, Sahlgrenska Academy, University of Gothenburg;; 2 Department of Surgery and Orthopaedics, Kungälv Hospital;; 3 The Swedish Hip Arthroplasty Register;; 4 Department of Orthopedics, Clinical Sciences, Lund University and Skåne University Hospital, Lund, Sweden;; 5 The Swedish Knee Arthroplasty Register

## Abstract

Background and purpose — Fast-track care programs in elective total hip and knee replacement (THR/TKR) have been introduced in several countries during the last decade resulting in a significant reduction of hospital stay without any rise in readmissions or early adverse events (AE). We evaluated the risk of readmissions and AE within 30 and 90 days after surgery when a fast-track program was introduced in routine care of joint replacement at 8 Swedish hospitals.

Patients and methods — Fast-track care programs were introduced at 8 public hospitals in Västra Götaland region from 2012 to 2014. We obtained data from the Swedish Hip and Knee Arthroplasty Registers for patients operated with THR and TKR in 2011–2015. All readmissions and new contacts with the health care system within 3 months with a possible connection to the surgical intervention were requested from the regional patient register. We compared patients operated before and after the introduction of the fast-track program.

Results — Implementation of the fast-track program resulted in a decrease in median hospital length of stay (LOS) from 5 to 3 days in both THR and TKR. The total readmission rate <90 days for THR was 7.2% with fast-track compared with 6.7% in the previous program, and for TKR 8.4% in both groups. Almost half of the readmissions occurred without any AE identified. There was no statistically significant difference concerning readmissions or AE when comparing the programs.

Interpretation — Implementation of a fast-track care program in routine care of elective hip and knee replacement is effective in reducing hospital stay without increasing the risk of readmissions or adverse events within 90 days after surgery.

Fast-track care programs have been introduced in elective joint replacement in several countries during the last decade (Antrobus and Bryson 2011, Raphael et al. 2011, Fawcett et al. 2012, Husted 2012, Okamoto et al. 2016). Inventors and pioneers of the fast-track concept have reported considerable reduction of hospital length-of-stay (LOS) and high patient satisfaction without increased readmissions or adverse events within 90 days (Larsen et al. 2008, Husted et al. 2010a and b, Machin et al. 2013, Glassou et al. 2014, Khan et al. 2014). A systematic review and meta-analysis of fast-track hip and knee arthroplasty (Zhu et al. 2017) concluded that the fast-track concept reduces LOS and the incidence of complications but does not appear to impact the 30-day readmission rate. However, most studies are observational cohort studies comparing the fast-track concept with historical data in providers dedicated to the new concept. Some uncertainty remains as to how to define fast-track as the care programs and clinical pathways are quite complex. The care processes in the cohorts not considered as fast-track differ in various aspects. Whilst LOS has gained much attention (Walters et al. 2016), one could argue for more focus on rapid recovery of function, and that short LOS should be balanced against any increase in morbidity and the cost of advanced follow-up (Thienpont et al. 2015, Lovecchio et al. 2016). One publication has reported a trend to higher infection rate after introducing the fast-track concept in THR (Amlie et al. 2016), but most studies are supporting equal or better results compared with conventional care in different outcome measures (Pilot et al. 2006, Husted et al. 2010a, b and c, Machin et al. 2013, Glassou et al. 2014, Khan et al. 2014, Stambough et al. 2015, Stowers et al. 2016, Delanois et al. 2017, Wilches et al. 2017, Zhu et al. 2017). It has been questioned whether the good results can be generalized and whether patient safety is ensured when the fast-track programs are broadly introduced in routine arthroplasty practice (Antrobus and Bryson 2011, Raphael et al. 2011, Jørgensen and Kehlet 2013). Our aim was to evaluate the risk of readmissions or adverse events (AE) within 30 and 90 days after surgery when a fast-track program was introduced in routine care of joint replacement in a defined region of Sweden.

## Patients and methods

To define the fast-track programs and the time of implementation a questionnaire was sent to hospitals performing elective hip and knee replacements in the Swedish Region Västra Götaland, a county council with a population of 1.7 million inhabitants. In 3 clinics without weekend service and exclusively patients with ASA 1–2, a care program based on the fast-track principles had already been implemented before 2011. These clinics were excluded from our study. In 8 public hospitals fast-track care programs were implemented between January 2012 and November 2014 at different times. We defined that fast-track was implemented when the following criteria for standard of care were met: (1) admission on the day of surgery, (2) mobilization within 3–6 hours after the operation, (3) functional discharge criteria in practice, and (4) an intended median length of stay (LOS) not more than 3 days. The patients were informed about the expected LOS, but the decision on discharge followed the functional ability and pain relief. However, regardless of whether the care program was defined as fast-track or not, the standard of care included written and oral structured information at a preoperative visit with a multiprofessional team 1–3 weeks before surgery, multimodal analgesia for pain relief, and tranexamic acid to reduce bleeding. Spinal anesthesia was routinely preferred supplemented by local infiltration analgesia in knee replacements but not in hips. 3 doses of cloxacillin were given on the day of surgery. The length of antithrombotic prophylaxis was 10 days in knees and 28–30 days in hips, but the antithrombotic drug varied between hospitals. No drains were used, a urinary catheter only in selected cases, and tourniquet in TKR was optional depending on the surgeon’s preference.

We collected data from the Swedish Knee and Hip Arthroplasty Registers and linked them to the regional patient register. In the 8 hospitals 7,774 elective THRs and 6,374 TKRs for osteoarthritis were performed between 2011 and 2015. The LOS was defined as the number of days by using the date of discharge minus the date of admission as the formula for calculation. Data on readmissions and new contacts within 30 and 90 days after surgery were retrieved from the regional patient register. Not only were outpatient contacts at the hospitals requested but also contacts with primary health care. Adverse events were defined based on International Classification of Diseases (ICD-10) codes for diagnoses and the Nordic Medico-Statistical Committee (NOMESCO) Classification of Surgical Procedures codes for interventions. The code list has been elaborated by the Swedish Knee Arthroplasty Register (SKAR) in collaboration with the National Board of Health and Welfare to be used after knee replacements. Based on the same principles we elaborated a code list adapted for elective hip replacements. It includes all local complications, secondary fractures, and tendon ruptures in the lower extremity, thromboembolic events, myocardial infarction, pneumonia, gastro-duodenal ulcers, acute kidney injury, and urinary retention (Appendices 1 and 2, see Supplementary data).

**Figure 1.**
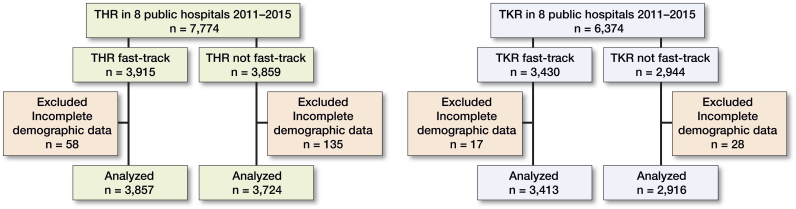
Patient allocation.

The patients were divided into 2 groups depending on whether they were operated in a fast-track program or not (Figure 1). There were 3,915 THRs and 3,430 TKRs in fast-track programs and 3,859 THRs and 2,944 TKRs in programs not considered as fast-track. No patients were excluded from the fast-track program after the implementation, and the intention was that all patients should follow the same clinical pathway and care program. If they stayed longer than 3 days, they were still included in the fast-track group.

## Statistics

Univariable and multivariable logistic regression analyses were used to evaluate the risk of readmissions and adverse events within 30 and 90 days. At low incidences of the outcome, odds ratios are good approximations for relative risks and can be interpreted as such (Davies et al. 1998). Relative risks were therefore approximated by odds ratios (OR) and estimated with a 95% confidence interval (CI). The results were considered statistically significant if observed p-values were smaller than 0.05. The statistical analyses were performed using R software package (version 3.4 or later; http://www.r-project.org) with the packages “tidyverse” (Hadley Wickham 2017) and “data.table” (Dowle and Srinivasan 2017).

## Ethics, funding, and potential conflicts of interest

The study was approved by the Regional Ethical Review Board in Gothenburg (Dnr 388-15, 2015-06-01 and 2015-07-17, T 1107-16, 2016-12-15). Financial support was received from the Healthcare Committee, Region Västra Götaland. No competing interests were declared.

## Results

Implementation of the fast-track program reduced LOS with statistical significance for both THR and TKR. The mean LOS for hips decreased from 5.8 days to 3.7 and from 5.4 to 3.3 for knees. All hospitals except 1 achieved a median value of LOS 3 days or less compared with 5 days or more in the previous care program. After implementation of fast-track the capacity increased at the public hospitals and included more ASA 1–2 patients, who previously had been sent to private clinics or hospitals accepting only ASA 1–2. This can explain why ASA 1–2 was slightly more frequent in the fast-track group. Demography, LOS, and surgical data are presented in Tables 1 and 2.

**Table 1.** Demographics on total hip replacement patients and data on operations. Values are frequency and (percentage) unless ­otherwise stated

	Not fast-track	Fast-track	
Factor	n = 3,859	n = 3,915	p-value
Patients with complete data	3,724	3,857	
Mean LOS, days	5.8	3.7	< 0.001
Median LOS, days (IQR)	5 (4–6)	3 (2–4)	
ASA 1–2	3,138 (84)	3,395 (88)	< 0.001
Age, mean (SD)	69.5 (10.3)	69.5 (10.1)	
Sex, female	2,186 (59)	2,200 (57)	0.2
BMI, mean (SD)	27.5 (4.6)	27.4 (4.6)	0.6
Posterolateral approach	1,021 (27)	1,441 (37)	
Direct lateral approach	2,688 (72)	2,396 (62)	
Cemented	2,582 (69)	2,578 (67)	
Uncemented	675 (18)	720 (19)	
Hybrid	150 (4.0)	271 (7.0)	
Reverse hybrid	317 (8.5)	288 (7.5)	

LOS: length of stay; IQR: interquartile range; ASA: American Society of Anesthesiologists; SD: standard deviation.

**Table 2.** Demographics on total knee replacement patients. Values are frequency and (percentage) unless ­otherwise stated

	Not fast-track	Fast-track	
Factor	n = 2,944	n = 3,430	p-value
Patients with complete data	2,916	3,413	
Mean LOS, days	5.4	3.3	< 0.001
Median LOS, days (IQR)	5 (4–6)	3 (2–4)	
Age, mean (SD)	69.5 (9.3)	68.8 (9.0)	0.002
Sex, female	1,734 (59)	1,952 (57)	0.08
ASA 1–2	2,493 (85)	3,035 (89)	< 0.001
BMI, mean (SD)	29.2 (4.9)	29.2 (4.60)	1.0

For abbreviations, see Table 1.

**Figure 2.**
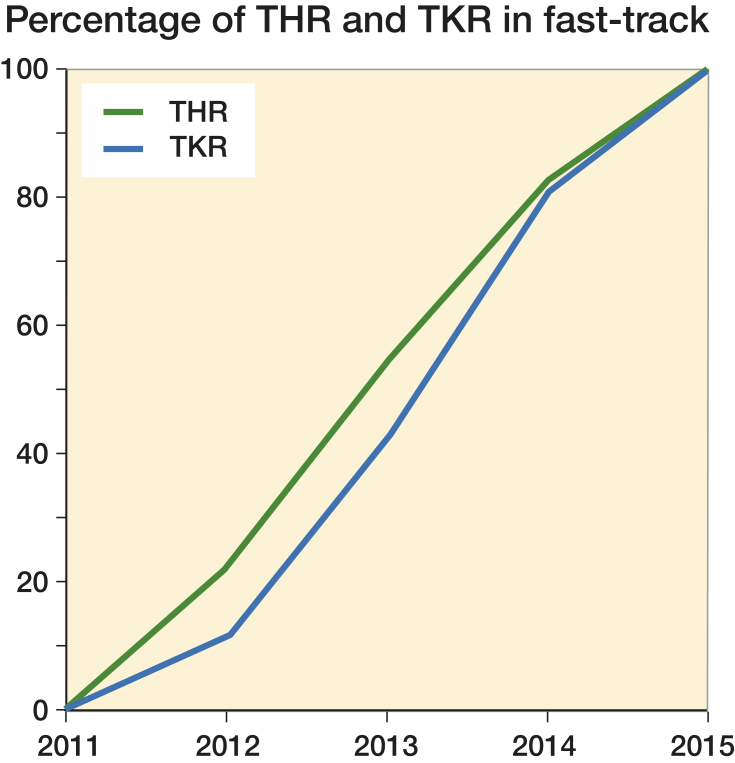
Percentage of THR and TKR in fast-track programs 2011–2015.

Fast-track care programs were implemented between January 2012 and November 2014 at different times in all 8 hospitals. Thus, in 2011 no patients were operated in the fast-track program and in 2015 all patients followed the program (Figure 2).

Most adverse events after surgery were identified at the hospital, either during the first hospital stay, at an unplanned readmission, or at a new contact as outpatient not necessarily related to the joint replacement. However, 10–15% of the new contacts due to an adverse event after surgery were identified at a primary health center outside the hospital. The numbers of patients with the first identified AE at different levels in the health care system are presented in Tables 3 and 4 and the total number of readmissions in Table 5. Almost half of the readmissions were due to other reasons; no AE could be identified in association with the readmission.

**Table 3.** First care contact for adverse events (AEs) after total hip replacement. Values are frequency and (percentage)

	Not fast-track	Fast-track
Factor	n = 3,859	n = 3,915
AE <30 days		
Primary health care	13 (0.3)	25 (0.6)
Hospital outpatient	82 (2.1)	82 (2.1)
Hospitalization	156 (4.0)	162 (4.1)
AE <90 days		
Primary health care	35 (0.9)	46 (1.2)
Hospital outpatient	110 (2.9)	107 (2.7)
Hospitalization	194 (5.0)	196 (5.0)

**Table 4.** First care contact for adverse events (AEs) after total knee replacement. Values are frequency and (percentage)

	Not fast-track	Fast-track
	n = 2,944	n = 3,430
AE <30 days		
Primary health care	17 (0.6)	29 (0.8)
Hospital outpatient	70 (2.4)	97 (2.8)
Hospitalization	126 (4.3)	140 (4.1)
AE <90 days		
Primary health care	29 (1.0)	54 (1.6)
Hospital outpatient	115 (3.9)	162 (4.7)
Hospitalization	156 (5.3)	173 (5.0)

**Table 5.** Total number of patients with readmissions <30 and <90 days after surgery. Values are frequency and (percentage)

	Not fast-track	Fast-track
THR, n	3,859	3,915
All readmissions <30 days	168 (4.4)	196 (5.0)
Readmissions <30 days with AE	97 (2.5)	111 (2.8)
All Readmissions <90 days	260 (6.7)	281 (7.2)
Readmissions <90 days with AE	141 (3.7)	151 (3.9)
TKR, n	2,944	3,430
All readmissions <30 days	159 (5.4)	193 (5.6)
Readmissions <30 days with AE	75(2.5%)	93 (2.7)
All readmissions <90 days	246 (8.4)	288 (8.4)
Readmissions <90 days with AE	120 (4.1)	139 (4.1)

Multivariable logistic regression showed no statistically significant influence of fast-track on readmission or adverse events. The OR of readmission after THR in the fast-track group was 1.2 (CI 0.9–1.5) within 30 days and 1.1 (CI 0.9–1.3) within 90 days. The OR of AE within 30 days in the fast-track group was 1.1 (CI 0.9–1.3) and 1.1 (0.9–1.2) within 90 days. For TKR the OR of readmission after TKA in the fast-track program was 1.1 (CI 0.9–1.4) within 30 days and 1.1 (CI 0.9–1.3) within 90 days. The OR of AE after TKA was estimated at 1.1 (CI 0.9–1.3) within 30 days and 1.2 (1.0–1.4) within 90 days.

The overall complication rate was similar regardless of whether the fast-track program was applied or not both for the major local and general complications (Tables 6 and 7). Pulmonary embolism was slightly more frequent in the fast-track group, particularly of TKR, but not statistically significant. The rates of clinical deep venous thrombosis (DVT) were almost the same. The number of patients with urinary retention was higher in the fast-track groups. We noticed that about 30% of them were treated at the health centers outside the hospitals.

**Table 6.** Adverse events <90 days after total hip replacement according to ICD-10 codes. Values are frequency and (percentage)

		Not fast-track	Fast-track
Factor	Codes	n = 3,859	n = 3,915
Deep infection**^a^**	T84.5	39 (1.0)	47 (1.2)
Surgical site infection	T81.4	58 (1.5)	42 (1.1)
Hip dislocation	Combinations	51 (1.3)	33 (0.8)
Myocardial infarction	I21	12 (0.3)	8 (0.2)
Cerebrovascular event	I60–I65	14 (0.4)	15 (0.4)
Deep venous thrombosis	I80	29 (0.8)	35 (0.9)
Pulmonary embolism	I26.0, I26.9	10 (0.3)	14 (0.4)
Acute kidney injury	N17, N99.0	9 (0.2)	9 (0.2)
Urinary retention	R33.9	9 (0.2)	28 (0.7)
Pneumonia	J15–J18	10 (0.3)	21 (0.5)
Gastrointestinal ulcers	K25–K27	13 (0.3)	15 (0.4)
Constipation	K59.0	11 (0.3)	11 (0.3)

**^a^**Prosthesis joint infection

**Table 7.** Adverse events <90 days after total knee replacement according to ICD-10 codes. Values are frequency and (percentage)

		Not fast-track	Fast-track
Factor	Codes	n = 2,944	n = 3,430
Deep infection**^a^**	T84.5	49 (1.7)	43 (1.3)
Surgical site infection	T81.4	32 (1.1)	42 (1.2)
Knee stiffness with manipulation NGT19	M24.5	16 (0.5)	18 (0.5)
Myocardial infarction	I21	9 (0.3)	11 (0.3)
Cerebrovascular event	I60–I65	12 (0.4)	13 (0.4)
Deep venous thrombosis	I80	38 (1.3)	45 (1.3)
Pulmonary embolism	I26.0, I26.9	8 (0.3)	21 (0.6)
Acute kidney injury	N17, N99.0	6 (0.2)	8 (0.2)
Urinary retention	R33.9	11 (0.4)	17 (0.5)
Pneumonia	J15–J18	14 (0.5)	25 (0.7)
Gastrointestinal ulcers	K25–K27	19 (0.6)	23 (0.7)
Constipation	K59.0	15 (0.5)	9 (0.3)

However, for most AEs the differences are small, and we abstain from statistical analysis as the diagnosis of the reported events cannot be confirmed by medical records.

In the fast-track group 8 patients (0.2%) died within 90 days after THR compared with 11 patients (0.3%) without fast-track program. 2 patients (0.1%) died within 90 days after TKR with fast-track and 6 patients (0.2%) with the previous care program.

## Discussion

We did not find any statistically significant increase in readmissions or adverse events within 30 and 90 days after surgery when a fast-track program was implemented in routine care of elective total hip and knee replacement (THR and TKR) at 8 Swedish hospitals. As expected, the mean LOS decreased by more than 2 days in both THR and TKR—a reduction of more than one-third. The absence of statistically significant difference in AE frequency or readmission is not an evidence of equivalence. However, concerns about fast-track surgery causing increased AEs and readmission were not substantiated in our results. The absolute differences in AE frequencies between groups were 0.3% or smaller.

A recent study from Finland presented an increased rate of readmissions in one hospital after implementation of a fast-track program (Pamilo et al. 2018). However, most other publications have reported unchanged rate of readmissions with fast-track total joint replacement (Husted et al. 2010b, Glassou et al. 2014, Khan et al. 2014).

One of the most severe and costly complications in joint replacement surgery is prosthetic joint infection (PJI). It has been questioned whether the fast-track program can increase the risk of PJI and the findings in a small sample from Norway (Amlie et al. 2016) raised concern leading to the program being abandoned after a short period. In contrast, a much larger study from Denmark (Glassou et al. 2014) reported that the risk of readmission due to infection may decrease over time after introduction of the new concept. In our study, the rate of PJI is slightly higher in the fast-track group for hips but lower for knees as defined by the ICD-10 code M845. The differences were not statistically significant. However, some uncertainty may remain regarding the real infection rate as the diagnosis is not confirmed by investigating medical records to know whether the criteria for PJI have been fulfilled. The aim of our study was to compare the groups and not to conclude the exact infection rate.

Among local complications, we found similar rates of knee stiffness requiring manipulation (0.5%) in the 2 groups. The manipulation rate is quite low, but longer follow-up may be needed (Husted et al. 2015). The rate of hip dislocation was low compared with other studies (Jorgensen et al. 2014) but probably reliable, as we have searched for all possible ICD-10 and NOMESCO codes in the regional patient register and not only readmissions. The rate was even lower in the fast-track cohort and we conclude that early discharge does not increase risk of hip dislocation.

It has been argued that early mobilization might reduce the risk of deep venous thrombosis (DVT) and the necessity of prolonged use of antithrombotic drugs has been questioned in the fast-track joint replacement setting (Husted et al. 2010c). In our study, reduction of symptomatic DVT among fast-track patients could not be demonstrated. Contrary to the expected, we found a higher number of pulmonary embolisms among TKR patients operated in the fast-track program although this difference was not statistically significant. Some uncertainty remains as the difference in mobilization regime between the 2 groups is not evident, the figures are quite low, and confirmation of the diagnosis in medical records is lacking.

The rates of pneumonia, gastro-duodenal ulcers, and acute kidney injury were similar in the 2 groups. Postoperative urinary retention is common (Bjerregaard et al. 2015) but if the problem is resolved before discharge, it would not be identified as a complication. The diagnosis code for urinary retention was uncommon in this Swedish context. However, a very short length of hospital stay may increase the need for a new contact with the health system, and the higher rate in the fast-track groups, especially in THR, may partly be explained by the fact that more than one-third of the patients with urinary retention were identified as new contacts in the primary health care system shortly after discharge.

The overall rates of readmissions and AE were similar without any statistical significant difference between the groups. Readmission rates and the incidence of AEs do not differ considerably from other studies (Husted et al. 2010b, Wolf et al. 2012, Zmistowski et al. 2013, Glassou et al. 2014) even if there are some differences in how the AEs are defined. The primary health care system was slightly more frequent as the first source of contact due to AEs in the fast-track group for both hips and knees but more than 85% of the AEs were identified and assessed at the hospitals as outpatients or inpatients. The AEs identified in the primary health care system were dominated by medical events.

The mortality rate within 30 and 90 days after THR and TKR is generally low with fewer deaths after implementation of the fast-track program, but as the numbers are small they do not allow reliable conclusions.

## Strengths and limitations

The strength of this study is that we have investigated routine care in arthroplasty surgery without patient selection. All hospitals had a defined date for the implementation of the fast-track according to our criteria. The national quality registers and patient register used have a very high completeness of data. The fact that also contacts in the primary health care system are included gives a more complete view of the short-term complications. However, we know that the coding in the health institutions is not always accurate. As we cannot confirm the accuracy of ICD-10 and NOMESCO codes by medical records some uncertainty remains concerning the correct incidence of specific complications. Another weakness is the difficulty in defining fast-track and in controlling all confounding factors in the clinical pathway and care process. The accuracy of LOS could be discussed as we had access only to the dates of admission and discharge and not the exact time point. Consequently, the LOS calculation may underestimate the real LOS if calculated by the hour, as the value of LOS is equal to the number of nights spent at the hospital. However, most publications report the LOS based on the same calculation (Husted et al. 2010a and b, Brock et al. 2017).

## Conclusion

Patient safety is preserved in the fast-track program but not better compared with other care programs for elective joint replacements in a Swedish context. To achieve reduction of adverse events more specific measures are needed.

## Supplementary data

Appendices 1 and 2 are available as supplementary data in the online version of this article, http://dx.doi.org/10.1080/17453674.2018.1492507

The authors would like to thank Daniel Odin for help with parts of the statistical analyses.

UB, MS, and OR conceived and planned the study. UB and EB performed the statistical analyses. All authors discussed the results and commented on the manuscript, which was drafted by UB.

*Acta* thanks Henrik Husted and other anonymous reviewers for help with peer review of this study.

## Supplementary Material

Supplemental Material
