# Supplementary material for: No increase in readmissions or adverse events after implementation of fast-track program in total hip and knee replacement at 8 Swedish hospitals: An observational before-and-after study of 14,148 total joint replacements 2011–2015
Source: Acta Orthop. 2018 Jul 9;89(5):522–7. doi: 10.1080/17453674.2018.1492507 (PMC6202734; doi:10.1080/17453674.2018.1492507)
Supplement: Supplemental Material [file IORT_A_1492507_SM9947.pdf]

# Supplementary data

## Appendix 1: ICD 10- and NOMESCO codes used for definition of adverse events according to the Swedish Knee Arthroplasty Register (SKAR)

### *DA. Surgical diagnosis*

**If the codes occur as the main or a secondary diagnosis during the first admission or as the main diagnosis during a later admission**

| Exact code | |
|---|---|
| G978 | Other postprocedural disorders of nervous system |
| G979 | Postprocedural disorder of nervous system, unspecified |
| M966G | Fracture of bone following insertion of orthopaedic implant, joint prosthesis, or bone plate knee/lower leg |
| M968 | Other postprocedural musculoskeletal disorders |
| M969 | Postprocedural musculoskeletal disorder, unspecified |
| T810 | Haemorrhage and haematoma complicating a procedure, not elsewhere classified |
| T812 | Accidental puncture and laceration during a procedure, not elsewhere classified |
| T813 | Disruption of operation wound, not elsewhere classified |
| T814 | Infection following a procedure, not elsewhere classified |
| T815 | Foreign body accidentally left in body cavity or operation wound following a procedure |
| T816 | Acute reaction to foreign substance accidentally left during a procedure |
| T817 | Vascular complications following a procedure, not elsewhere classified |
| T818 | Other complications of procedures, not elsewhere classified |
| T818W | Other complications of procedures, not elsewhere classified |
| T819 | Unspecified complication of procedure |
| T840 | Mechanical complication of internal joint prosthesis |
| T840G | Mechanical complication of internal joint prosthesis knee/lower leg |
| T843 | Mechanical complication of other bone devices, implants and grafts |
| T843G | Mechanical complication of other bone devices, implants and grafts knee/lower leg |
| T844 | Mechanical complication of other internal orthopaedic devices, implants and grafts |
| T844G | Mechanical complication of other internal orthopaedic devices, implants and grafts, knee/lower leg |
| T845 | Infection and inflammatory reaction due to internal joint prosthesis |
| T845G | Infection and inflammatory reaction due to internal joint prosthesis knee/lower leg |
| T847 | Infection and inflammatory reaction due to other internal orthopaedic prosthetic devices, implants and grafts |
| T847G | Infection and inflammatory reaction due to other internal orthopaedic prosthetic devices, implants and grafts knee/lower leg |
| T848 | Other complications of internal orthopaedic prosthetic devices, implants and grafts |
| T848G | Other complications of internal orthopaedic prosthetic devices, implants and grafts knee/lower leg |
| T849 | Unspecified complication of internal orthopaedic prosthetic device, implant and graft |
| T888 | Other specified complications of surgical and medical care, not elsewhere classified |
| T889 | Complication of surgical and medical care, unspecified |

### *DB. Diagnosis for knee-related events*

**If the codes occur as the main or a secondary diagnosis during the first admission or as a main diagnosis during a later admission**

| Exact code | |
|---|---|
| G573 | Lesion of lateral popliteal nerve |
| G574 | Lesion of medial popliteal nerve |
| M000 | Staphylococcal arthritis and polyarthritis |
| M000G | Staphylococcal arthritis and polyarthritis, knee |
| M002G | Other streptococcal arthritis and polyarthritis, knee |
| M008G | Arthritis and polyarthritis due to other specified bacterial agents, knee |
| M009G | Pyogenic arthritis, unspecified, knee |
| M220 | Recurrent dislocation of patella |
| M221 | Recurrent subluxation of patella |
| M236 | Other spontaneous disruption of ligament(s) of knee |
| M244G | Recurrent dislocation and subluxation of joint, knee |
| M621G | Other rupture of muscle (nontraumatic), knee/lower leg |
| M662G | Spontaneous rupture of extensor tendons, knee/lower leg |
| M663G | Spontaneous rupture of flexor tendons, knee/lower leg |
| M843G | Stress fracture, lower leg |
| S342 | Injury of nerve root of lumbar and sacral spine |
| S800 | Contusion of knee |
| S810 | Open wound of knee |
| S830 | Dislocation of patella |
| S831 | Dislocation of knee |
| S834L | Sprain and strain involving lateral collateral ligament of knee |
| S834M | Sprain and strain involving medial collateral ligament of knee |
| S835R | Sprain and strain involving anterior cruciate ligament of knee |
| S835S | Sprain and strain involving anterior cruciate ligament of knee |
| S835X | Sprain and strain involving cruciate ligament of knee, unspecified |
| S840 | Injury of tibial nerve at lower leg level |
| S841 | Injury of peroneal nerve at lower leg level |

**If the codes occur as the main diagnosis after the first admission**

| Exact code | |
|---|---|
| M235 | Chronic instability of knee |
| M240G | Loose body in joint, knee |
| M245G | Contracture of joint, knee |
| M246G | Ankylosis of joint, knee |
| M256 | Stiffness of joint, not elsewhere classified |
| M256G | Stiffness of joint, not elsewhere classified, knee |
| M659G | Synovitis and tenosynovitis, unspecified, knee |
| M860G | Acute haematogenous osteomyelitis, knee/lower leg |
| M861G | Other acute osteomyelitis, knee/lower leg |
| M866 | Other chronic osteomyelitis |
| M866G | Other chronic osteomyelitis, knee/lower leg |
| M895G | Osteolysis, knee/lower leg |

## *DC. Cardiovascular diagnosis*

**If the codes occur as the main or a secondary diagnosis during the first admission or as the main diagnosis during a later admission**

| Exact code | |
|---|---|
| I260 | Pulmonary embolism with mention of acute cor pulmonale |
| I269 | Pulmonary embolism without mention of acute cor pulmonale |
| I460 | Cardiac arrest with successful resuscitation |
| I461 | Sudden cardiac death, so described |
| I469 | Cardiac arrest, unspecified |
| I490 | Ventricular fibrillation and flutter |
| I649 | Stroke, not specified as haemorrhage or infarction |
| I770 | Arteriovenous fistula, acquired |
| I771 | Stricture of artery |
| I772 | Rupture of artery |
| I819 | Portal vein thrombosis |
| I978 | Other postprocedural disorders of circulatory system, not elsewhere classified |
| I979 | Postprocedural disorder of circulatory system, unspecified |
| J809 | Adult respiratory distress syndrome |
| J819 | Pulmonary oedema |
| T811 | Shock during or resulting from a procedure, not elsewhere classified |

**Code starts with**

| | |
|---|---|
| I21 | Acute myocardial infarction |
| I24 | Other acute ischaemic heart diseases |
| I60 | Subarachnoid haemorrhage |
| I61 | Intracerebral haemorrhage |
| I62 | Other nontraumatic intracranial haemorrhage |
| I63 | Cerebral infarction |
| I65 | Occlusion and stenosis of precerebral arteries, not resulting in cerebral infarction |
| I66 | Occlusion and stenosis of cerebral arteries, not resulting in cerebral infarction |
| I72 | Other aneurysm and dissection |
| I74 | Arterial embolism and thrombosis |
| I82 | Other venous embolism and thrombosis |

## *DM. Diagnoses for other medical events*

**If the codes occur as the main or a secondary diagnosis during the first admission or as a secondary diagnosis during a later admission**

| Exact code | |
|---|---|
| J952 | Acute pulmonary insufficiency following nonthoracic surgery |
| J953 | Chronic pulmonary insufficiency following surgery |
| J955 | Postprocedural subglottic stenosis |
| J958 | Other postprocedural respiratory disorders |
| J959 | Postprocedural respiratory disorder, unspecified |
| J981 | Pulmonary collapse |
| N990 | Postprocedural renal failure |
| N998 | Other postprocedural disorders of genitourinary system |
| N999 | Postprocedural disorder of genitourinary system, unspecified |
| R339 | Retention of urine |

**Code starts with**

| | |
|---|---|
| L89 | Decubitus ulcer and pressure area |
| I80 | Phlebitis and thrombophlebitis |
| J13 | Pneumonia due to Streptococcus pneumoniae |
| J14 | Pneumonia due to Haemophilus influenzae |
| J15 | Bacterial pneumonia, not elsewhere classified |
| J16 | Pneumonia due to other infectious organisms, not elsewhere classified |
| J17 | Pneumonia in diseases classified elsewhere |
| J18 | Pneumonia, organism unspecified |
| J96 | Respiratory failure, not elsewhere classified |
| K25 | Gastric ulcer |
| K26 | Duodenal ulcer |
| K27 | Peptic ulcer, site unspecified |
| N17 | Acute renal failure |

**If the codes occur as the main diagnosis after the first admission**

| Exact code | |
|---|---|
| K590 | Constipation |
| N991 | Postprocedural urethral stricture |

**Code starts with**

| | |
|---|---|
| J20 | Acute bronchitis |
| J21 | Acute bronchiolitis |
| J22 | Unspecified acute lower respiratory infection |
| K29 | Gastritis and duodenitis |

## *A. Surgical intervention codes*

**If the codes occur during the first admission at a date after the primary surgery date or as the main intervention code at a later date**

| Exact code | |
|---|---|
| NFQ09 | Exarticulation of hip |
| NFQ19 | Amputation of femur |
| NFQ99 | Other amputation or related operation on hip or femur |
| NGB59 | Patellofemoral prosthesis, supplementary |
| NGF01 | Total synovectomy of knee, arthroscopic |
| NGF02 | Total synovectomy of knee, open |
| NGF10 | Partial synovectomy of knee, percutaneous |
| NGF11 | Partial synovectomy of knee, arthroscopic |
| NGF12 | Partial synovectomy of knee, open |
| NGF91 | Other operation on synovia or joint surface of knee, arthroscopic |
| NGF92 | Other operation on synovia or joint surface of knee, open |
| NGK09 | Excision of fragment of bone of knee or lower leg |
| NGK19 | Partial or total excision of bone of knee or lower leg |
| NGM09 | Fasciotomy of knee or lower leg |
| NGQ09 | Exarticulation of knee |
| NGT09 | Removal of foreign body from tissue of knee or lower leg |
| NGT19 | Forcible manipulation of knee joint |
| QDA10 | Incision of skin of lower limb. Includes: Drainage |
| QDB00 | Suture of skin of lower limb |
| QDB05 | Revision of wound of skin of lower limb |
| QDB99 | Other procedure for wound of skin of lower limb |
| QDE35 | Repair of skin defect of lower limb after surgery or injury |
| TNG05 | Incision of soft tissue, knee and lower leg |
| TNG10 | Arthrocentesis, knee joint |

**Code starts with**

| | |
|---|---|
| NGA | Exploratory procedures on knee and lower leg |
| NGC | Secondary prosthetic replacement of knee joint |
| NGE | Operations on capsules and ligaments of knee joint |
| NGG | Excision, reconstruction and fusion of knee joint |
| NGH | Miscellaneous operations on knee joint. Includes: Reduction of dislocations |
| NGJ | Fracture surgery of knee and lower leg |
| NGL | Operations on muscles and tendons of knee and lower leg |
| NGS | Operations for infection of tendons, joints and bone of knee and lower leg |
| NGU | Removal of implants and external fixation devices from knee and lower leg |
| NGW | Reoperations on knee and lower leg |
| QDG | Operations for chronic ulcers and fistulae of skin of lower limb |

 *Acta Orthopaedica* 2018; 89 (DOI: 10.1080/17453674.2018.1492507)

# Appendix 2: ICD 10- and NOMESCO codes used for definition of adverse events according to the Swedish Hip Arthroplasty Register (SHAR)

## *DA. Surgical diagnosis*

**If the codes occur as the main or a secondary diagnosis during the first admission or as the main diagnosis during a later admission**

| Exact code | |
|---|---|
| G978 | Other postprocedural disorders of nervous system |
| G979 | Postprocedural disorder of nervous system, unspecified |
| M966F | Fracture of bone following insertion of orthopaedic implant, joint prosthesis, or bone plate hip/femur |
| M968 | Other postprocedural musculoskeletal disorders |
| M969 | Postprocedural musculoskeletal disorder, unspecified |
| T810 | Haemorrhage and haematoma complicating a procedure, not elsewhere classified |
| T812 | Accidental puncture and laceration during a procedure, not elsewhere classified |
| T813 | Disruption of operation wound, not elsewhere classified |
| T814 | Infection following a procedure, not elsewhere classified |
| T815 | Foreign body accidentally left in body cavity or operation wound following a procedure |
| T816 | Acute reaction to foreign substance accidentally left during a procedure |
| T817 | Vascular complications following a procedure, not elsewhere classified |
| T818 | Other complications of procedures, not elsewhere classified |
| T818W | Other complications of procedures, not elsewhere classified |
| T819 | Unspecified complication of procedure |
| T840 | Mechanical complication of internal joint prosthesis |
| T840F | Mechanical complication of internal joint prosthesis hip/femur |
| T843 | Mechanical complication of other bone devices, implants and grafts |
| T843F | Mechanical complication of other bone devices, implants and grafts hip/femur |
| T844 | Mechanical complication of other internal orthopaedic devices, implants and grafts |
| T844F | Mechanical complication of other internal orthopaedic devices, implants and grafts, hip/femur |
| T845 | Infection and inflammatory reaction due to internal joint prosthesis |
| T845F | Infection and inflammatory reaction due to internal joint prosthesis hip/femur |
| T847 | Infection and inflammatory reaction due to other internal orthopaedic prosthetic devices, implants and grafts |
| T847F | Infection and inflammatory reaction due to other internal orthopaedic prosthetic devices, implants and grafts hip/femur |
| T848 | Other complications of internal orthopaedic prosthetic devices, implants and grafts |
| T848F | Other complications of internal orthopaedic prosthetic devices, implants and grafts hip/femur |
| T849 | Unspecified complication of internal orthopaedic prosthetic device, implant and graft |
| T888 | Other specified complications of surgical and medical care, not elsewhere classified |
| T889 | Complication of surgical and medical care, unspecified |

## *DB. Diagnosis for hip-related events*

**If the codes occur as the main or a secondary diagnosis during the first admission or as a main diagnosis during a later admission**

| Exact code | |
|---|---|
| G570 | Lesion of sciatic nerve |
| G571 | Meralgia paraesthetica |
| G572 | Lesion of femoral nerve |
| M000 | Staphylococcal arthritis and polyarthritis |
| M000F | Staphylococcal arthritis and polyarthritis hip/femur |
| M002F | Other streptococcal arthritis and polyarthritis hip/femur |
| M008F | Arthritis and polyarthritis due to other specified bacterial agents hip/femur |
| M009F | Pyogenic arthritis, unspecified hip/femur |
| M243 | Pathological dislocation and subluxation of joint, not elsewhere classified |
| M244 | Recurrent dislocation and subluxation of joint |
| M244F | Recurrent dislocation and subluxation of joint hip/femur |
| S730 | Dislocation of hip |

| Code starts with | |
|---|---|
| S74 | Injury of nerves at hip and thigh level |
| S75 | Injury of femoral artery |
| S76 | Injury of muscle and tendon of hip |

**If the codes occur as the main diagnosis after the first admission**

| Exact code | |
|---|---|
| M240F | Loose body in joint hip/femur |
| M245F | Contracture of joint hip/femur |
| M246F | Ankylosis of joint hip/femur |
| M610F | Myositis ossificans traumatica |
| M614 | Other calcification of muscle |
| M614F | Other calcification of muscle hip/femur |
| M621F | Other rupture of muscle (nontraumatic) hip/femur |
| M662F | Spontaneous rupture of extensor tendons hip/femur |
| M663F | Spontaneous rupture of flexor tendons hip/femur |
| M843F | Stress fracture, hip/femur |
| M860F | Acute haematogenous osteomyelitis hip/femur |
| M861F | Other acute osteomyelitis hip/femur |
| M866 | Other chronic osteomyelitis hip/femur |
| M866F | Other chronic osteomyelitis hip/femur |
| M895E | Osteolysis pelvis |

## *DC. Cardiovascular diagnosis*

**If the codes occur as the main or a secondary diagnosis during the first admission or as the main diagnosis during a later admission**

| Exact code | |
|---|---|
| I260 | Pulmonary embolism with mention of acute cor pulmonale |
| I269 | Pulmonary embolism without mention of acute cor pulmonale |
| I460 | Cardiac arrest with successful resuscitation |
| I461 | Sudden cardiac death, so described |
| I469 | Cardiac arrest, unspecified |
| I490 | Ventricular fibrillation and flutter |
| I649 | Stroke, not specified as haemorrhage or infarction |
| I770 | Arteriovenous fistula, acquired |
| I771 | Stricture of artery |
| I772 | Rupture of artery |
| I819 | Portal vein thrombosis |
| I978 | Other postprocedural disorders of circulatory system, not elsewhere classified |
| I979 | Postprocedural disorder of circulatory system, unspecified |
| J809 | Adult respiratory distress syndrome |
| J819 | Pulmonary oedema |
| T811 | Shock during or resulting from a procedure, not elsewhere classified |

**Code starts with**

| | |
|---|---|
| I21 | Acute myocardial infarction |
| I24 | Other acute ischaemic heart diseases |
| I60 | Subarachnoid haemorrhage |
| I61 | Intracerebral haemorrhage |
| I62 | Other nontraumatic intracranial haemorrhage |
| I63 | Cerebral infarction |
| I65 | Occlusion and stenosis of precerebral arteries, not resulting in cerebral infarction |
| I66 | Occlusion and stenosis of cerebral arteries, not resulting in cerebral infarction |
| I72 | Other aneurysm and dissection |
| I74 | Arterial embolism and thrombosis |
| I82 | Other venous embolism and thrombosis |

## *DM. Diagnoses for other medical events*

**If the codes occur as the main or a secondary diagnosis during the first admission or as a secondary diagnosis during a later admission**

| Exact code | |
|---|---|
| J952 | Acute pulmonary insufficiency following nonthoracic surgery |
| J953 | Chronic pulmonary insufficiency following surgery |
| J955 | Postprocedural subglottic stenosis |
| J958 | Other postprocedural respiratory disorders |
| J959 | Postprocedural respiratory disorder, unspecified |
| J981 | Pulmonary collapse |
| N990 | Postprocedural renal failure |
| N998 | Other postprocedural disorders of genitourinary system |
| N999 | Postprocedural disorder of genitourinary system, unspecified |
| R339 | Retention of urine |

**Code starts with**

| | |
|---|---|
| L89 | Decubitus ulcer and pressure area |
| I80 | Phlebitis and thrombophlebitis |
| J13 | Pneumonia due to Streptococcus pneumoniae |
| J14 | Pneumonia due to Haemophilus influenzae |
| J15 | Bacterial pneumonia, not elsewhere classified |
| J16 | Pneumonia due to other infectious organisms, not elsewhere classified |
| J17 | Pneumonia in diseases classified elsewhere |
| J18 | Pneumonia, organism unspecified |
| J96 | Respiratory failure, not elsewhere classified |
| K25 | Gastric ulcer |
| K26 | Duodenal ulcer |
| K27 | Peptic ulcer, site unspecified |
| N17 | Acute renal failure |

**If the codes occur as the main diagnosis after the first admission**

| Exact code | |
|---|---|
| K590 | Constipation |
| N991 | Postprocedural urethral stricture |

**Code starts with**

| | |
|---|---|
| J20 | Acute bronchitis |
| J21 | Acute bronchiolitis |
| J22 | Unspecified acute lower respiratory infection |
| K29 | Gastritis and duodenitis |

 *Acta Orthopaedica* 2018; 89 (DOI: 10.1080/17453674.2018.1492507)

## *B. Surgical intervention codes*

**If the codes occur during the first admission at a date after the primary surgery date or as the main intervention code at a later date**

| Exact code | |
|---|---|
| NFQ09 | Exarticulation of the hip joint |
| NFA02 | Exploration of soft tissue of hip and thigh, open surgery |
| NFA11 | Diagnostic arthroscopy of hip joint |
| NFA12 | Exploration of hip joint, open surgery |
| NFA20 | Biopsy of soft tissue or joint of hip and thigh, percutaneous |
| NFA21 | Biopsy of soft tissue or joint of hip and thigh, arthroscopic |
| NFA22 | Biopsy of soft tissue or joint of hip and thigh, open |
| QDA10 | Incision of skin of lower limb. Includes: Drainage |
| QDE35 | Repair of skin defect of lower limb after surgery or injury |
| TNF05 | Incision of soft tissue hip and thigh |
| TNF10 | Arthrocentesis of hip joint |
| QDB00 | Suture of skin of lower limb |
| QDB05 | Revision of wound of skin of lower limb |
| QDB99 | Other procedure for wound of skin of lower limb |

**Code starts with**

| | |
|---|---|
| NFC | Secondary prosthetic replacement of hip joint |
| NFF | Operations on synovia and surfaces of hip joint |
| NFG | Excision, reconstruction and fusion of hip joint |
| NFH | Miscellaneous operations on hip joint. Includes: Reduction of dislocations |
| NFJ | Fracture surgery of femur |
| NFK | Operations on femur |
| NFL | Operations on muscles and tendons of hip and thigh |
| NFM | Operations on muscles and tendons of hip and thigh |
| NFS | Operations for infection of tendons, joints and bone of hip and thigh |
| NFT | Miscellaneous operations on hip or thigh |
| NFU | Removal of implants and external fixation devices from hip and femur |
| NFW | Reoperations on hip or thigh |
| QDG | Operations for chronic ulcers and fistulae of skin of lower limb |
